# Heat transfer flow of Maxwell hybrid nanofluids due to pressure gradient into rectangular region

**DOI:** 10.1038/s41598-020-73174-1

**Published:** 2020-10-06

**Authors:** Yu-Ming Chu, Rizwan Ali, Muhammad Imran Asjad, Ali Ahmadian, Norazak Senu

**Affiliations:** 1 Department of Mathematics, Huzhou University, Huzhou, 313000 People’s Republic of China; 2 Hunan Provincial Key Laboratory of Mathematical Modeling and Analysis in Engineering, Changsha University of Science and Technology, Changsha, 410114 People’s Republic of China; 3 Department of Mathematics, University of Management and Technology, Lahore, Pakistan; 4 Institute of IR 4.0, The National University of Malaysia, UKM, 43400 Bangi, Selangor Malaysia; 5 School of Mathematical Sciences, College of Science and Technology, Wenzhou-Kean University, Wenzhou, China; 6 Institute for Mathematical Research, University Putra Malaysia (UPM), 43400 Serdang, Selangor Malaysia

**Keywords:** Mathematics and computing, Applied mathematics

## Abstract

In this work, influence of hybrid nanofluids (Cu and $\mathrm{Al}_{2}\mathrm{O}_{3}$) on MHD Maxwell fluid due to pressure gradient are discussed. By introducing dimensionless variables the governing equations with all levied initial and boundary conditions are converted into dimensionless form. Fractional model for Maxwell fluid is established by Caputo time fractional differential operator. The dimensionless expression for concentration, temperature and velocity are found using Laplace transform. As a result, it is found that fluid properties show dual behavior for small and large time and by increasing volumetric fraction temperature increases and velocity decreases respectively. Further, we compared the Maxwell, Casson and Newtonian fluids and found that Newtonian fluid has greater velocity due to less viscosity. Draw the graphs of temperature and velocity by Mathcad software and discuss the behavior of flow parameters and the effect of fractional parameters.

## Introduction

In industry and engineering many physical methods exist who have incomplete viscoelastic fluid, most common of these are molten plastics, synthetic propellants, exotic lubricants, suspension solutions, polymer solutions food stuffs, and so many other examples of viscoelastic fluid. These fluids have been modeled in a number of different behaviors with their constitutive equations varying greatly in complexity, among which the viscoelastic Maxwell fluid model has been studied widely Fetecau and Fetecau^1^, Tan and Masuoka^2^, Jamil et al.^3^ and Abbasbandy et al.^4^. Christensen^5^ give the Maxwell model can be represented by a purely viscous damper and a purely elastic spring connected in series, which has been proposed to describe the behavior of viscoelastic fluids, and has some success in describing polymeric liquids, it being more amenable to analysis and more importantly experimental. Rheological constitutive equations with fractional derivatives Podlubny^6^, Song and Jiang^7^ and Imran et al.^8^ have been proved to be a valuable tool to describe the behaviors of viscoelastic properties. The fractional derivative models of the viscoelastic fluids are derived from classical equations, which are modified by replacing the time derivative of an integer order by precisely non-integer order integrals or derivatives. Song and Jiang^7^ for the analysis of viscoelastic gum, experimental data used the fractional calculus method and by this method more reliable results were gained. Fetecau et al.^9^ studied the unsteady fluid flow of a second-grade cause by the time-dependent motion of a plate between two side walls perpendicular to the plate. Xue et al.^10^ and Xue and Nie^11^ discussed the Rayleigh Stokes problem and find out the solutions by heating the viscoelastic fluid in a porous half-space. Jamil et al.^3^ find out the irregular flow of an condensed Maxwell fluid in which fractional derivative were produced by a sudden moved plate, and find out the effect on fluid motion by fractional limits and by materials. Qi and Guo^12^ studied a new equation based on heat conduction and that equation was based on time-nonlocal generalized of Fourier law, the perfect solution of an initial-boundary value problem was studied and presented by series forms. Fan et al.^13^ introduced a converse issue to find out parameters in establishing fractional Zener model based on the Bayesian method, and for the justification of the method some examples were performed. Imran et al.^8^ investigated differnent fluids and find out their convection flow by using Caputo fractional derivatives, and by finding the fluid velocity using the Laplace transform method.

Magnetohydrodynamics (MHD) is the study of the behavior of electrically conducting fluids, i.e. a plasma or some other collection of charged particles, in a magnetic field. The collective motion of the particles gives rise to an electric field that interacts with the magnetic field and causes the plasma motion to alter. This coupling between hydrodynamic forces and magnetic forces means that the magnetic field is effectively ‘frozen into’ the plasma; the field lines flow with the plasma, and can be stretched, squeezed, or looped. One consequence is that the frozen-in field lines of two plasmas prevent them from mixing. MHD has contributed to the understanding of the solar wind and its interaction with planetary magnetospheres, of solar flares and prominence. It was assumed that a liquid bond to a solid boundary and that condition called no-slip boundary is proved insufficient in many cases such as the mechanics of thin fluids. The large number of models have been proposed to explain the slip that on solid boundaries. In recent years, Zheng et al.^14^ find out the exact solutions of generalized Oldroyd-B fluid flow with the slip things. Han et al.^15^ presented a slip flow of a generalized Burger’s fluid between two side walls generalized by an exponential accelerating plate and a constant pressure, the analytical solutions are established and analyzed. Akbar and Khan^16^ given the numerical study of carbon nanotubes postponed magnetohydrodynamics (MHD) stagnation point flow over a stretching sheet with convective slip. Shakeel et al.^17^ studied the flows of an Oldroyd-B fluid under the consideration of slip condition at the boundary, the fluid motion is generated by the flat plate which has a translational motion in its plane with a time-dependent velocity. Hayat et al.^18^ find out the unstable flow of magnetohydrodynamics (MHD) over stretching sheet with velocity and thermal slip boundary conditions, and many different boundaries were find out on to calculate velocity and temperature. Ji et al.^19^ report on Dirac monopoles with a polar-core vortex induced by spin-orbit coupling in ferromagnetic Bose-Einstein condensates. Ji et al.^20^ working at three-dimensional study of the ring vortex solitons is conducted for both attractive and repulsive Bose-Einstein condensates subject to harmonic potential confinement. The localized nonlinear matter waves of the quasi-two-dimensional Bose-Einstein condensates with spatially modulated nonlinearity in the harmonic potential investigated by Shan et al.^21^. Wen et al.^22^ study the matter rogue wave in Bose–Einstein condensates with attractive interatomic interaction analytically and numerically. Fei et al.^23^ working at the crystallized (triangular, square, honeycomb) and amorphous vortices in rotating atomic-molecular Bose-Einstein condensates (BECs) by using the damped projected Gross-Pitaevskii equation. Fei et al.^24^ explore the rotating spin-1 Bose-Einstein condensates with anisotropic spin-orbit coupling by using the damped projected Gross-Pitaevskii equation. Some other references on Bose-Einstein condensates can be seen in^25–28^.

Hybird materials were defined by Yamada et al.^29^ as combination of two or more than two constituents at molecular level and out of these two substances one is inorganic and other is organic, for example the covalent of bonds between silanol molecular inorganic / organic hybrids and polymers. Makishma^30^ divided the substances in three groups according to their chemical modes (i.e. metals). Baghbanzadeh et al.^31^ find out the position of rheological properties of water based nanofluids and multi wall carbon nanotubes (MWCNTs). By a new designed concept of Niihara^32^ exhibited the nanoparticles that enhanced thermal and mechanical properties. The things discussed above are primarily based on experimental study of hybrid nanoparticles. Since then, a few more practical studies have been done in this area. Iqbal et al.^33^ find the rotating oscillating vertical channel of the hybrid nanofluids. They supposed hall current thermal radiation with three different shapes of nanoparticles. They discovered that the platelet shapes of hybrid nanoparticles and heat transfer augments with volume fraction are found to have the highest temperature.

In complex dynamics, many cases of physical sciences and engineering cannot represent the classical or integer order derivative. Fractional calculus plays an important role in signal handling, chemical reactions, biomedical sciences, viscoelastic flows etc. the integer order derivative in fractional calculus is interchanged with non integer order derivative will show the characteristics of memory influence of flow. In literature fractional models can be create in power law model, fractional statistical models, fluid dynamics, geophysics, fractional wavelet model^34,36,37^. Vieru et al.^38^ by using the concept of Caputo time fractional derivatives studied the time fractional free convection flow of a generalized viscous fluid. Khan et al.^39^ using the Caputo fractional operator to made the model of Casson fluid .

The above researchers does not find the hybrid Maxwell nanofluid due to pressure gradient. In this work we find the effect of hybird nanofluids (Cu and $\mathrm{Al}_{2}\mathrm{O}_{3}$) on MHD Maxwell nanofluid due to pressure gradient and this is a new trend. The governing equations are obtained by introducing the dimensionless variables. Caputo time fractional derivative operator developed fractional model of hybrid Maxwell nanofluids with sodium alginate base fluid. Due to higher thermal conductivities Copper and Aluminium Oxide are considered as the nanoparticles. With the help of Laplace transform to find the solutions of temperature and velocity. The inverse Laplace transform are obtained by using Stehfest’s and Tzou’s algorithmic. Using Mathcad’s software analytical solutions are designed graphically for fractional and flow parameters.

## Statement of the problem

**Figure 1:**
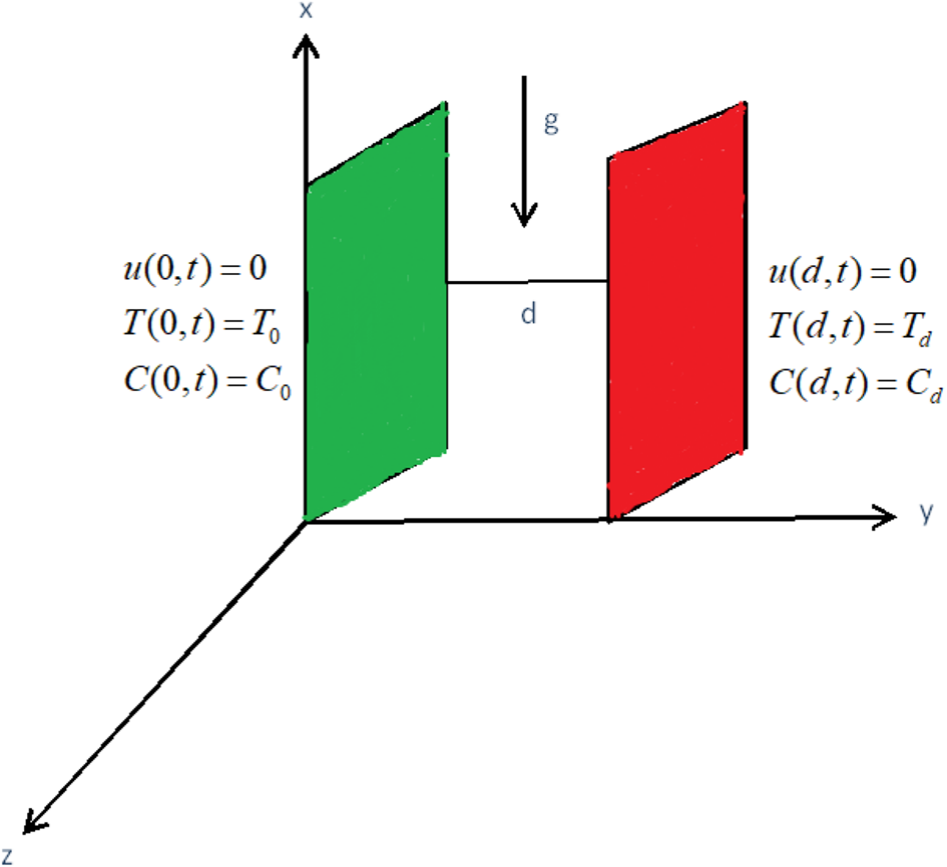
Physical model and Coordinate system.

Let the unsteady flow of sodium alginate based hybrid nanofluid (Cu and $\mathrm{Al}_{2}\mathrm{O}_{3}$) in a vertical channel. Let the distance d between two parallel plates. The x-axis is taken along one of the plate which is fixed in the vertically upward direction and y-axis is normal to the plate. Initially, at time $t = 0$, both the plates and the fluid are considered to be at the temperature $T_d$. At time $t > 0$, the temperature of the fluid at $y = 0$ is raised to $T_o$, causing the flow of free convection currents as shown in Fig. 1.

The governing equations are

- The balance of linear momentum equation in the absence of body force is given by
  1 $$\begin{aligned} \rho _ {hbnf}\,\,\partial _{t}\, u(y,t)= & - \frac{\partial p}{\partial x} \,+ \, \partial _{y} \tau _{1}(y,t)+ \,\, (T- T_{0})\,g \,(\rho \beta _ {T})_{hbnf} \nonumber \\&\quad + (C- C_{0})\,g \,(\rho \beta _ {c})_{hbnf} - \sigma _{_{hbnf}} B_{0}^{2} \ u(y,t), \end{aligned}$$
- The constitutive relation for Maxwell fluid is given by
  2 $$\begin{aligned} \left( 1 + \lambda _{1}\,\partial _{t}\right) \tau _{1} (y, t) - \mu _ {hbnf} \partial _{y} u(y, t) = 0, \end{aligned}$$
- The equation of thermal balance
  3 $$\begin{aligned} (\rho C_{p}) _ {hbnf}\,\, \partial _{t} T(y,t) = - \partial _{y} q_{1} (y, t), \end{aligned}$$
- The thermal flux equation find by Fourier’s law of heat conduction by Haristov^40^ and Povstenko^41^.
  4 $$\begin{aligned} q_{1}(y,t) + k_{hbnf} \partial _{y} T (y, t) = 0, \end{aligned}$$
- The equation of diffusion balance is
  5 $$\begin{aligned} \partial _{t} C (y, t)= - \partial _{y} J_{1}(y, t), \end{aligned}$$
- The equation of molecular diffusion is
  6 $$\begin{aligned} J_{1}(y, t) + D \partial _{y} C(y, t) = 0, \end{aligned}$$

where $u = u(y,t)$, $T = T(y,t)$, $C = C(y,t)$, $\rho _{hbnf}$, $\mu _{hbnf}$, $\sigma _{hbnf}$, $\beta _{T}$, $\beta _{C}$, *g*, $(\rho c_{p})_{hbnf}$, $k_{hbnf}$ and $D_{hbnf}$ are respectively fluid velocity in the x-direction, temperature, concentration, density, the dynamic viscosity, electrical conductivity of the base fluid, volumetric thermal expansion coefficient, gravitational acceleration, heat capacitance of nanofluids, thermal conductivity of nanofluid and thermal diffusion coefficient.

Appropriate initial and boundary conditions are:

7 $$\begin{aligned} u(y,0)= & 0,\,\, u(0,t) = 0,\,\,\,\, u(d,t) = 0,\ \ 0\le d\le 1, \end{aligned}$$

8 $$\begin{aligned} T(y,0)= & T_{0},\,\, T(0,t) = T_{0},\,\, T(d,t) = T_{d}, \end{aligned}$$

9 $$\begin{aligned} C(y,0)= & C_{0},\,\, C(0,t) = C_{0},\,\, C(d,t) = C_{d}. \end{aligned}$$

These relations are taken from^35^.

$$\begin{aligned} (\rho \beta )_{hbnf}= & (1 - \phi _{2})\,\,(\rho \beta )_{f}\left\{ (1 - \phi _{1}) + \phi _{1} \left( \frac{(\rho \beta )_{s1}}{(\rho \beta )_{f}}\right) \right\} + \phi _{2}\,\, (\rho \beta )_{s2}\,,\\&\mu _{hbnf} = \frac{\mu _{f}}{(1 - \phi _{2})^{2.5}(1 - \phi _{1})^{2.5}}, \\ (\rho c_{p}) _ {hbnf}= & (1 - \phi _{2})\,\,(\rho c_{p})_{f}\left\{ (1 - \phi _{1}) + \phi _{1} \left( \frac{(\rho c_{p})_{s1}}{(\rho c_{p})_{f}}\right) \right\} + \phi _{2}\,\,(\rho c_{p})_{s2}, \\ k_{hbnf}= & \left\{ \frac{k_{s2} + (s - 1)k_{bf} - (s - 1)\phi _{2}(k_{bf} - k_{s2})}{k_{s2} + (s - 1)k_{bf} + \phi _{2}(k_{bf} - k_{s2}}\right\} k_{bf}, \\ k_{bf}= & \left\{ \frac{k_{s1} + (s - 1)k_{f} - (s - 1)\phi _{1}(k_{f} - k_{s1})}{k_{s1} + (s - 1)k_{f} + \phi _{1}(k_{f} - k_{s1}}\right\} k_{f}. \end{aligned}$$

where $\phi _{1}$ and $\phi _{2}$ are the nanoparticles volume fraction, $\rho _{f}$, $\rho _{s1}$ and $\rho _{s2}$ are the density of the base fluid and hybrid nanoparticles, $\beta _{s1}$, $\beta _{s2}$ and $\beta _{f}$ are the volumetric coefficient of thermal expansions of nanoparticles and base fluids, $(C_{p})_{s1}$, $(C_{p})_{s2}$ and $(C_{p})_{f}$ are the specific heat capacities of nanoparticles and base fluids at constant pressure. Here $k_{f}$, $k_{s1}$ and $k_{s2}$ are thermal conductivities of base fluid and nanoparticles.

Introducing the non-dimensional variables and functions

$$\begin{aligned} u^{*}= & \frac{u}{U_{0}}, \,\,\,\, x^{*} = \frac{x}{d}, \,\,\,\, t^{*} = \frac{t U_{0}}{d}, \,\,\,\, y^{*} = \frac{y}{d}, \,\,\,\, p^{*} = \frac{d}{\mu U_{0}} p,\\ \theta= & \frac{T - T_{0}}{T_{d} - T_{0}}, C^{*} = \frac{C - C_{0}}{C_{d} - C_{0}},\ \omega ^{*} = \frac{\omega d}{U_{0}}, \\&\quad -\frac{\partial p^{*}}{\partial x^{*}} = \lambda _{0}^{*} + \lambda ^{*}\,\, exp (\textit{i}\, \omega ^{*} t^{*}),\,\,\,\, \tau _{1}^{*} = \frac{\tau _{1}}{\tau _{0}},\,\,\,\, q_{1}^{*} = \frac{q_{1}}{q_{0}},\,\,\,\, J_{1}^{*} = \frac{J_{1}}{J_{0}}. \end{aligned}$$

into Eqs. (1)–(6) and ignore the star notation.

10 $$\begin{aligned}&a_{1}\, Re\,\partial _{t} u(y, t) = H(t) \left\{ \lambda _{0} + \lambda \, exp (\textit{i}\, \omega \, t)\right\} + L \, \partial _{y} \tau _{1} (y, t) \nonumber \\&\quad + a_{2}\, \textit{Gr}\,\,\theta (y,t) + a_{3}\, \textit{Gm}\,\,C (y,t) - Mu(y,t), \end{aligned}$$

11 $$\begin{aligned}&(1 + \lambda _{2}\,\partial _{t}) \tau _{1} (y,t) - a_{0}\, \partial _{y} u(y,t) = 0, \end{aligned}$$

12 $$\begin{aligned}&\partial _{t} \theta (y,t) = - r_{1}\,\partial _{y} q_{1}(y,t), \end{aligned}$$

13 $$\begin{aligned}&q_{1}(y,t) + b_{0}\,\partial _{y} \theta (y,t) = 0, \end{aligned}$$

14 $$\begin{aligned}&\partial _{t} C(y,t) = - r_{2}\,\partial _{y} J_{1}(y,t), \end{aligned}$$

15 $$\begin{aligned}&J_{1}(y,t) + c_{0}\,\, \partial _{y} C(y,t) = 0, \end{aligned}$$

with dimensionless conditions

16 $$\begin{aligned} u(y,0)= & 0\,,\, u(0,t) = 0\,,\, u(1,t) = 0, \end{aligned}$$

17 $$\begin{aligned} \theta (y,0)= & 0\,,\, \theta (0,t) = 0\,,\, \theta (1,t) = 1, \end{aligned}$$

18 $$\begin{aligned} C(y,0)= & 0\,,\, C(0,t) = 0\,,\, C(1,t) = 1, \end{aligned}$$

where

$$\begin{aligned} a_{1}= & (1 - \phi _{2})\left\{ (1 - \phi _{1}) + \phi _{1} \frac{\rho _{s1}}{\rho _{f}}\right\} + \phi _{2}\frac{\rho _{s2}}{\rho _{f}}, \\ a_{2}= & \left[ (1 - \phi _{2})\,\,\left\{ (1 - \phi _{1}) + \phi _{1} \left( \frac{(\rho \beta _{T})_{s1}}{(\rho \beta _{T})_{f}}\right) \right\} + \phi _{2}\,\,\left( \frac{(\rho \beta _{T})_{s2}}{(\rho \beta _{T})_{f}}\right) \right] , \\ a_{3}= & \left[ (1 - \phi _{2})\,\,\left\{ (1 - \phi _{1}) + \phi _{1} \left( \frac{(\rho \beta _{c})_{s1}}{(\rho \beta _{c})_{f}}\right) \right\} + \phi _{2}\,\,\left( \frac{(\rho \beta _{c})_{s2}}{(\rho \beta _{c})_{f}}\right) \right] , \\ a_{4}= & \left[ (1 - \phi _{2})\,\,\left\{ (1 - \phi _{1}) + \phi _{1} \left( \frac{(\rho C_{p})_{s1}}{(\rho C_{p})_{f}}\right) \right\} + \phi _{2}\,\,\left( \frac{(\rho C_{p})_{s2}}{(\rho C_{p})_{f}}\right) \right] , \\ \text {Re}= & \frac{U_{0}\, d}{\nu }, \,\,\,\,\,\, L = \frac{d\tau _{0}}{\mu U_{0}},\,\,\,\,\,\, \lambda _{2} = \frac{\lambda _{1}\,U_{0}}{d},\,\,\,\,\,\, \text {Gr} = \frac{g\,\beta _{T}\,d^{2} (T_{d} - T_{0})}{\nu \,U_{0}}, \\&\quad \text {Gm} = \frac{g\,\beta _{c}\,d^{2} (C_{d} - C_{0})}{\nu \,U_{0}},\,\,\,\,\,\, \text {Pr} = \frac{(\mu c_{p})_{f}}{k_{f}}, \\ a_{0}= & \frac{\mu _{hbnf} \,\,U_{0}}{\tau _{0}\,\, d},\,\,\,\,\,\, b_{0} = K_{hbnf} \left( \frac{T_{d} - T_{0}}{q_{0} d} \right) ,\\&\quad c_{0} = D \left( \frac{C_{d} - C_{0}}{J_{0} d}\right) ,\,\,\,\,\,\, r_{1} = \frac{q_{0}}{(\rho c_{p})_{f} U_{0}(T_{d} - T_{0})a_{4}}, \\ r_{2}= & \frac{J_{0}}{U_{0} (C_{d} - C_{0})},\,\,\,\, M = \frac{\sigma \,B_{0}^{2}\,d^{2}}{\mu _{f}}. \end{aligned}$$

### Basic definitions and fractional model

A generalized model of the classical constitutive relation of Maxwell fluid for shear stress by using the concept of Blair and Caffyn^43^.

19 $$\begin{aligned} (1 + \lambda _{2}\,\partial _{t}) \tau _{1}(y,t) = a_{1 - \alpha }\,\, ^{C}D_{t}^{1-\alpha }\left\{ \frac{\partial u(y,t)}{\partial y}\right\} ,\,\,\,\,\,\,\,\, 0\,<\,\alpha \, \le \, 1, \end{aligned}$$

Clearly when relaxation parameter $\lambda _{2}=0$, we get the generalized constitutive relation for Newtonian fluid.

Hristov^40^ and Povstenko^41^ find the constitutive thermal flux equation generalized Fourier’s law

20 $$\begin{aligned} q_{1} (y,t) = - b_{1 - \beta }\,\, ^{C}D_{t}^{1-\beta }\left\{ \frac{\partial \theta (y,t)}{\partial y}\right\} ,\,\,\,\,\,\,\,\, 0\,<\,\beta \, \le \, 1. \end{aligned}$$

The constitutive equation for diffusion balance equation by Fick’s law

21 $$\begin{aligned} J_{1}(y,t) = - c_{1 - \gamma }\,\, ^{C}D_{t}^{1-\gamma }\left\{ \frac{\partial C(y,t)}{\partial y}\right\} ,\,\,\,\,\,\,\,\, 0\,<\,\gamma \, \le \, 1 \end{aligned}$$

In the above relations $\alpha$, $\beta$ and $\gamma$ are fractional parameters and $^{C}D_{t}^{\alpha }$ is Caputo time fractional operator defined as^44,45^ where

$h_{\alpha }(t) = \frac{t^{-\alpha }}{\Gamma (1-\alpha )}$ is the singular power-law kernal, $g' (y,s) = \frac{\partial g(y,t)}{\partial t}\mid _{t=s}$ and $c_{1-\alpha }, d_{1-\beta }$, $e_{1-\gamma }$ are the generalized material coefficients.

For $\alpha , \beta , \gamma = 1$ reduce to the material coefficients $c_{0},d_{0}$ and $e_{0}$. The Laplace transform of Caputo time fractional operator is

22 $$\begin{aligned} L \{ ^{C}D_{t}^{\alpha } g(y, t) \} = s^{\alpha } L\left\{ g(y, s)\right\} - s^{\alpha -1} g(y,0), \end{aligned}$$

where ’L’ is the Laplace operator and is defined in^46^.

By using Eqs. (19), (20) and (21) into Eqs. (10), (12) and (14) the fractional differential equation of the mathematical model will be:

23 $$\begin{aligned}&a_{1}\, Re\,\,(1 + \lambda _{2}\,\frac{\partial }{\partial t})\,\, \partial _{t} u(y, t) = H(t) \left\{ \lambda _{0} + \lambda \, exp (\textit{i}\, \omega \, t)\right\} \nonumber \\&\quad + L\partial _{y}\left\{ a_{1 - \alpha }\,\, ^{C}D_{t}^{1-\alpha } \partial _{y} u(y,t)\right\} + (1 + \lambda _{2}\frac{\partial }{\partial t})\nonumber \\&\quad a_{2}\,\textit{Gr}\,\theta (y,t) + (1 + \lambda _{2}\frac{\partial }{\partial t})\,a_{3}\,\textit{Gm}\,C(y,t) - M\,(1 + \lambda _{2}\,\frac{\partial }{\partial t})\,u(y,t), \end{aligned}$$

24 $$\begin{aligned}&\partial _{t} \theta (y,t) = - P_{1} \partial _{y} \left\{ - b_{1 - \beta }\,\, ^{C}D_{t}^{1-\beta } \partial _{y} \theta (y,t)\right\} , \end{aligned}$$

25 $$\begin{aligned}&\partial _{t} C(y,t) = - P_{2} \partial _{y} \left\{ - c_{1 - \gamma }\,\, ^{C}D_{t}^{1-\gamma } \partial _{y} C (y,t)\right\} . \end{aligned}$$

We apply left inverse operators $I_{t}^{1-\alpha }(.), I_{t}^{1-\beta }(.)$ and $I_{t}^{1-\gamma }(.)$ to Eqs. (23), (24) and (25)

26 $$\begin{aligned}&a_{1}\, Re\,\,(1 + \lambda _{2}\,\frac{\partial }{\partial t})\,\, I_{t}^{1-\alpha } \partial _{t} u(y, t) = H(t) \left\{ \lambda _{0} + \lambda \, exp (\textit{i}\, \omega \, t)\right\} \nonumber \\&\quad + L\,a_{1 - \alpha } \partial _{y}^{2}u(y,t) + (1 + \lambda _{2}\frac{\partial }{\partial t})a_{2}\,\textit{Gr}\nonumber \\&\quad I_{t}^{1-\alpha } \theta (y,t) + (1 + \lambda _{2}\frac{\partial }{\partial t})\,\,a_{3}\,\textit{Gm}\,\,I_{t}^{1-\alpha } C (y,t)\nonumber \\&\quad - M\,(1 + \lambda _{2}\,\frac{\partial }{\partial t})\,\,I_{t}^{1-\alpha }u(y,t), \end{aligned}$$

27 $$\begin{aligned}&I_{t}^{1-\beta } \partial _{t} \theta (y,t) = r_{1} b_{1-\beta } \partial _{y}^{2} \theta (y,t), \end{aligned}$$

28 $$\begin{aligned}&I_{t}^{1-\gamma } \partial _{t} C(y,t) = r_{2} c_{1 - \gamma }\,\, \partial _{y}^{2} C (y,t). \end{aligned}$$

or equivalently

29 $$\begin{aligned}&a_{1}\, Re\,\, (1 + \lambda _{2}\,\frac{\partial }{\partial t})\,\,^{c}D_{t}^{\alpha } u(y, t) = H(t) \left\{ \lambda _{0} + \lambda \, exp (\textit{i}\, \omega \, t)\right\} + L a_{1 - \alpha } \partial _{y}^{2}u(y,t) + (1 + \lambda _{2}\,\frac{\partial }{\partial t})\,\,\nonumber \\&\quad a_{2}\,\textit{Gr}\,I_{t}^{1-\alpha } \theta (y,t) + (1 + \lambda _{2}\,\frac{\partial }{\partial t})\, \,a_{3}\,\textit{Gm}\,\,I_{t}^{1-\alpha } C (y,t)- M\,(1 + \lambda _{2}\,\frac{\partial }{\partial t})\,\,I_{t}^{1-\alpha }u(y,t), \end{aligned}$$

30 $$\begin{aligned}&^{c}D_{t}^{\alpha } \theta (y,t) = r_{1} b_{1-\beta } \partial _{y}^{2} \theta (y,t), \end{aligned}$$

31 $$\begin{aligned}&^{c}D_{t}^{\gamma } C(y,t) = r_{2} c_{1 - \gamma }\,\, \partial _{y}^{2} C (y,t).\nonumber \\&\quad Note: I_{t}^{1-\alpha } \partial _{t} u(y, t) = ^{c}D_{t}^{\alpha } u(y,t). \end{aligned}$$

## Solution of the problem

In this section we find the solution of the initial and boundary value problem given in Eqs. (29)–(31) with the help of Laplace transform.

The solution of Eq. (31) subject to boundary conditions $(18)_{2}-(18)_{3}$ with the help of Laplace transform technique.

32 $$\begin{aligned} {\overline{C}} (y,s) = \frac{1}{s}\left\{ \sum _{n=0}^{\infty } e^{\sqrt{\frac{s^{\gamma }}{p_{\gamma }}}(1+2n-y)} - \sum _{n=0}^{\infty } e^{\sqrt{\frac{s^{\gamma }}{p_{\gamma }}}(1+2n+y)}\right\} , \end{aligned}$$

where $$p_{\gamma } = r_{2}c_{1-\gamma }$$, for $$\gamma \rightarrow 1, p_{\gamma } = r_{2}c_{0} = \frac{1}{Sc}$$

The solution of Eq. (30) subject to boundary conditions $(17)_{2}-(17)_{3}$ with the help of Laplace transform technique.

33 $$\begin{aligned} {\overline{\theta }} (y,s) = \frac{1}{s}\left\{ \sum _{n=0}^{\infty } e^{\sqrt{\frac{s^{\beta } }{p_{\beta }}}\,(1\,+ 2n\,-y)} - \sum _{n=0}^{\infty } e^{\sqrt{\frac{s^{\beta }}{p_{\beta }}}\,(1\,+2n+\,y)}\right\} , \end{aligned}$$

where $$p_{\beta } = r_{1}b_{1-\beta }$$, for $$\beta \rightarrow 1, p_{\beta } = r_{1}b_{0} = \frac{1}{\textit{pr}\,Re\,a_{4}} . \frac{k_{hbnf}}{k_{f}}$$

The solution of Eq. (29) subject to boundary conditions $(16)_{2}-(16)_{3}$ with the help of Laplace transform technique.

34 $$\begin{aligned}&{\overline{u}}(y,s) = \left\{ \frac{1}{(1+\lambda _{2}s)(a_{1} Re s + M)}\right\} \left\{ \frac{\lambda _{0}}{s^{\alpha }} + \frac{\lambda s^{1-\alpha }}{s- \iota \omega }\right\} \left\{ -1 + e^{-\sqrt{\frac{\left( 1 + \lambda _{2}\,s\right) + (a_{1} Re s + M)}{p_{\alpha } s^{1-\alpha }}}}\right\} \nonumber \\&\left\{ \frac{e^{y\sqrt{\frac{\left( 1 + \lambda _{2}\,s\right) + (a_{1} Re s + M)}{p_{\alpha } s^{1-\alpha }}}}- e^{-y\sqrt{\frac{\left( 1 + \lambda _{2}\,s\right) + (a_{1} Re s + M)}{p_{\alpha } s^{1-\alpha }}}}}{e^{\sqrt{\frac{\left( 1 + \lambda _{2}\,s\right) + (a_{1} Re s + M)}{p_{\alpha } s^{1-\alpha }}}}-e^{-\sqrt{\frac{\left( 1 + \lambda _{2}\,s\right) + (a_{1} Re s + M)}{p_{\alpha } s^{1-\alpha }}}}}\right\} \nonumber \\&\quad - \frac{(1 + \lambda _{2} s) \,a_{2} \text {Gr} P_{\beta }}{s \left\{ P_{\beta } a_{1} Re s \left( 1 + \lambda _{2} s\right) + M P_{\beta }\left( 1 + \lambda _{2} s\right) - P_{\alpha } s^{1-\alpha +\beta } \right\} } \nonumber \\&\left\{ \sum _{n=0}^{\infty } e^{\sqrt{\frac{s^{\beta }}{p_{\beta }}}(2n)} - \sum _{n=0}^{\infty } e^{\sqrt{\frac{s^{\beta }}{p_{\beta }}}(2n+2)}\right\} \nonumber \\&\quad \left\{ \frac{e^{y\sqrt{\frac{\left( 1 + \lambda _{2}\,s\right) + (a_{1} Re s + M)}{p_{\alpha } s^{1-\alpha }}}}- e^{-y\sqrt{\frac{\left( 1 + \lambda _{2}\,s\right) + (a_{1} Re s + M)}{p_{\alpha } s^{1-\alpha }}}}}{e^{\sqrt{\frac{\left( 1 + \lambda _{2}\,s\right) + (a_{1} Re s + M)}{p_{\alpha } s^{1-\alpha }}}}-e^{-\sqrt{\frac{\left( 1 + \lambda _{2}\,s\right) + (a_{1} Re s + M)}{p_{\alpha } s^{1-\alpha }}}}}\right\} \nonumber \\&-\frac{(1 + \lambda _{2} s) \,a_{3} \text {Gm} P_{\gamma }}{s \left\{ P_{\gamma } a_{1} Re s \left( 1 + \lambda _{2} s\right) + M P_{\gamma }\left( 1 + \lambda _{2} s\right) - P_{\alpha } s^{1-\alpha +\gamma } \right\} }\left\{ \sum _{n=0}^{\infty } e^{\sqrt{\frac{s^{\gamma }}{p_{\gamma }}}(2n)} - \sum _{n=0}^{\infty } e^{\sqrt{\frac{s^{\gamma }}{p_{\gamma }}}(2n+2)}\right\} \nonumber \\&\left\{ \frac{e^{y\sqrt{\frac{\left( 1 + \lambda _{2}\,s\right) + (a_{1} Re s + M)}{p_{\alpha } s^{1-\alpha }}}}- e^{-y\sqrt{\frac{\left( 1 + \lambda _{2}\,s\right) + (a_{1} Re s + M)}{p_{\alpha } s^{1-\alpha }}}}}{e^{\sqrt{\frac{\left( 1 + \lambda _{2}\,s\right) + (a_{1} Re s + M)}{p_{\alpha } s^{1-\alpha }}}}-e^{-\sqrt{\frac{\left( 1 + \lambda _{2}\,s\right) + (a_{1} Re s + M)}{p_{\alpha } s^{1-\alpha }}}}}\right\} \nonumber \\&\quad +\left\{ \frac{1}{(1+\lambda _{2}s)(a_{1} Re s + M)}\right\} \left\{ \frac{\lambda _{0}}{s^{\alpha }} + \frac{\lambda s^{1-\alpha }}{s- \iota \omega }\right\} \nonumber \\&\left\{ 1 - e^{-\sqrt{\frac{\left( 1 + \lambda _{2}\,s\right) + (a_{1} Re s + M)}{p_{\alpha } s^{1-\alpha }}}}\right\} \nonumber \\&\quad + \frac{(1 + \lambda _{2} s) \,a_{2} \text {Gr} P_{\beta }}{s \left\{ P_{\beta } a_{1} Re s \left( 1 + \lambda _{2} s\right) + M P_{\beta }\left( 1 + \lambda _{2} s\right) - P_{\alpha } s^{1-\alpha +\beta } \right\} } \nonumber \\&\left\{ \sum _{n=0}^{\infty } e^{\sqrt{\frac{s^{\beta }}{p_{\beta }}}(1+2n-y)} - \sum _{n=0}^{\infty } e^{\sqrt{\frac{s^{\beta }}{p_{\beta }}}(1+2n+y)}\right\} \nonumber \\&\quad + \frac{(1 + \lambda _{2} s) \,a_{3} \text {Gm} P_{\gamma }}{s \left\{ P_{\gamma } a_{1} Re s \left( 1 + \lambda _{2} s\right) + M P_{\gamma }\left( 1 + \lambda _{2} s\right) - P_{\alpha } s^{1-\alpha +\gamma } \right\} } \nonumber \\&\left\{ \sum _{n=0}^{\infty } e^{\sqrt{\frac{s^{\gamma }}{p_{\gamma }}}(1+2n-y)} - \sum _{n=0}^{\infty } e^{\sqrt{\frac{s^{\gamma }}{p_{\gamma }}}(1+2n+y)}\right\} . \end{aligned}$$

where $p_{\alpha } = L \,a_{1-\alpha }$, for $\alpha \rightarrow 1, p_{\alpha } = L \,a_{0} = \frac{1}{(1 - \phi _{2})^{2.5} (1 - \phi _{1})^{2.5}}.$ Due to the complex nature of the problem, we are unable to find inverse Laplace transform. Therefore, for obtaining more accurate solution we applied some well known formulae to find inverse Laplace transform numerically.

The inverse Laplace transform of Eqs. (32)–(34) will be attained numerically by applying Tzou’s and Stehfest’s algorithms^47,48^.

### Numerical results and discussion

In the present paper we discuss the hybrid Maxwell nanofluid in a rectangular region under the outcome of magnetohydrodynamics and pressure gradient. By applying Laplace transform method satisfying all initial and boundary conditions, this model has been solved analytically. For the influence of different parameters, concentration, temperature and velocity of the hybrid nanofluid are graphically discussed. By using Stehfest’s algorithm and Tzou’s algorithm to find the inverse Laplace transform and verify our obtained results. For graphical presentation, the thermophysical properties for base fluid and nanoparticles are taken from Table 1.

**Table 1:** Thermophysical properties of nanofluids.

Physical properties	$\rho ( \frac{km}{m^{3}})$	$c_{p} (\frac{1}{kg\,\,k})$	$\sigma (\frac{s}{m})$	$k\,(\frac{W}{m\,k})$	$\beta \times 10^{5} (\frac{1}{k})$
Sodium Alginate	989	4175	$5.5 *10^{-6}$	0.6376	21
Copper $\phi _{1}$	8933	385	$59.6 *10^{6}$	400	1.67
Alumina $\phi _{2}$	3970	765	$35 *10^{6}$	40	0.85

**Figure 2:**
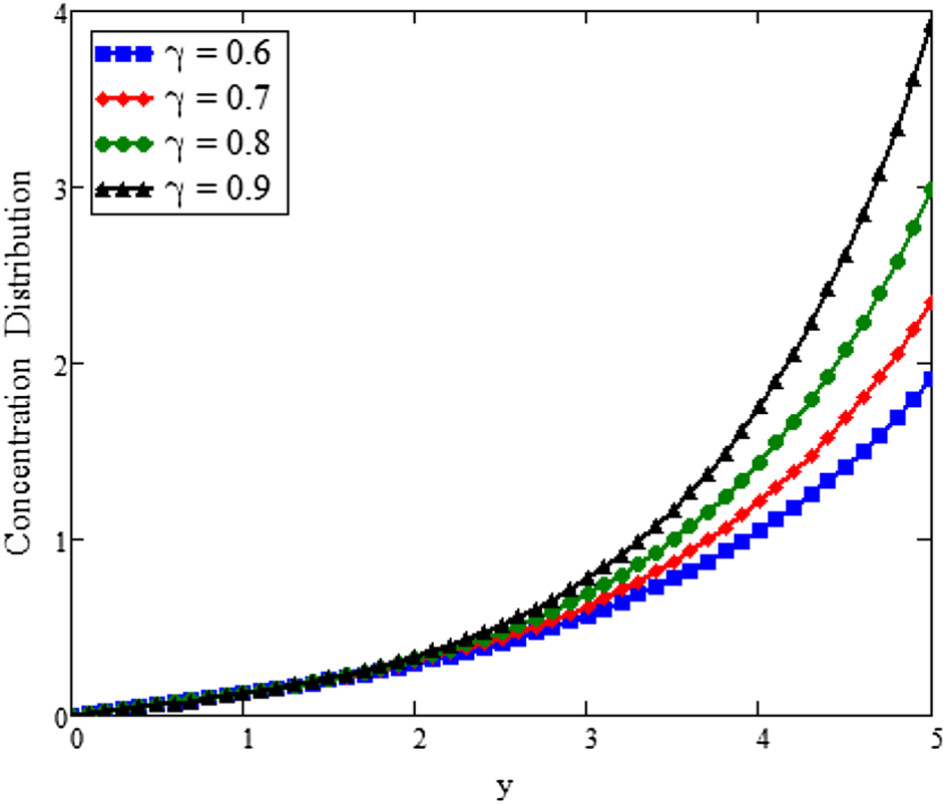
Concentration distribution against y due to $\gamma$ for two dimensional graph, when: $t=3$ and $Sc=6$.

**Figure 3:**
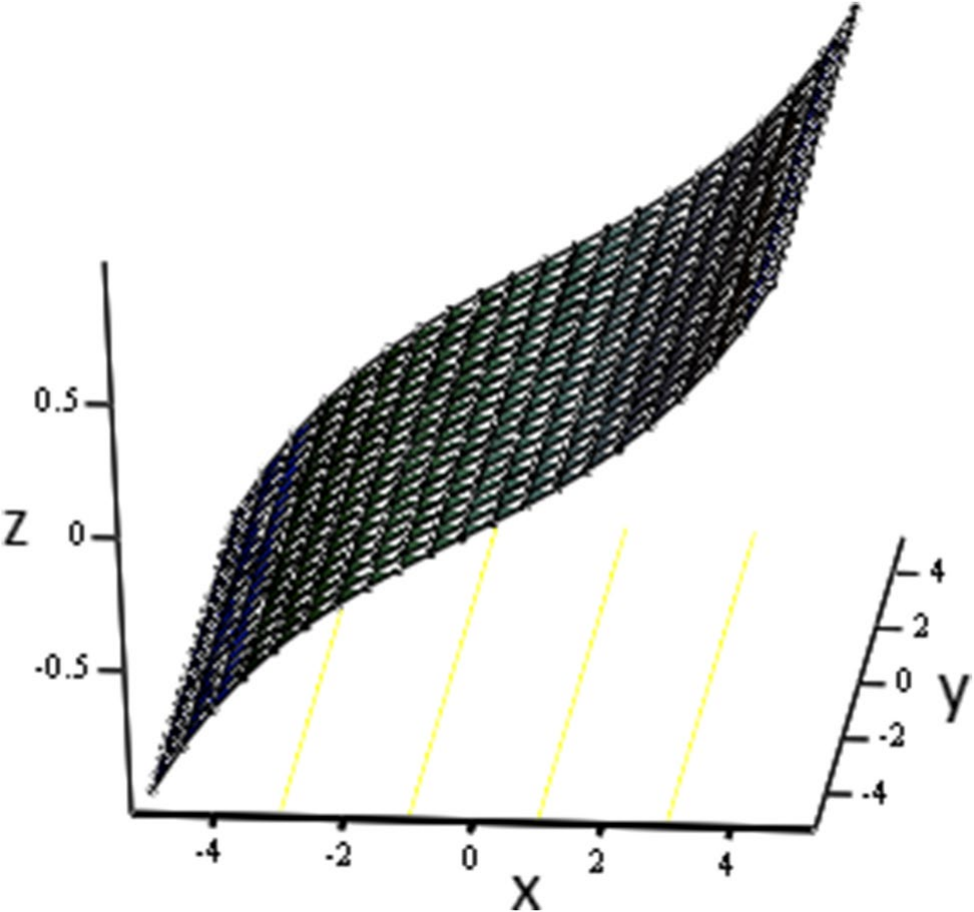
Concentration distribution against y due to $\gamma$ for three dimensional graph, when: $t=3$, $Sc=6$ and $\gamma =0.6$.

**Figure 4:**
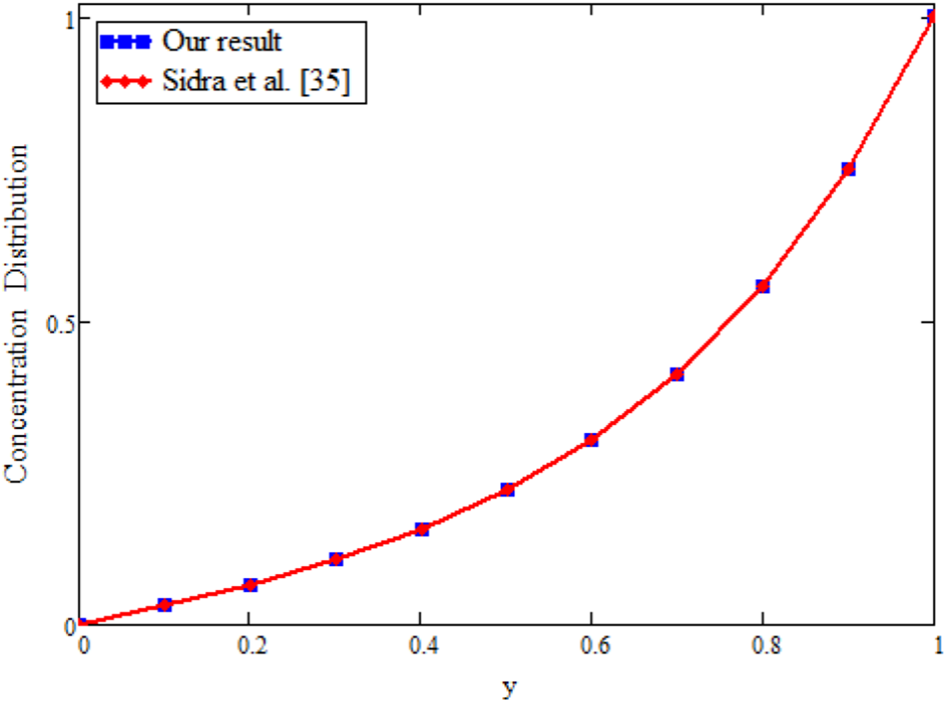
Concentration comparison of our result and Sidra et al.^35^, when: $t=0.1$, $\gamma =0.2$ and $Sc=6$.

**Figure 5:**
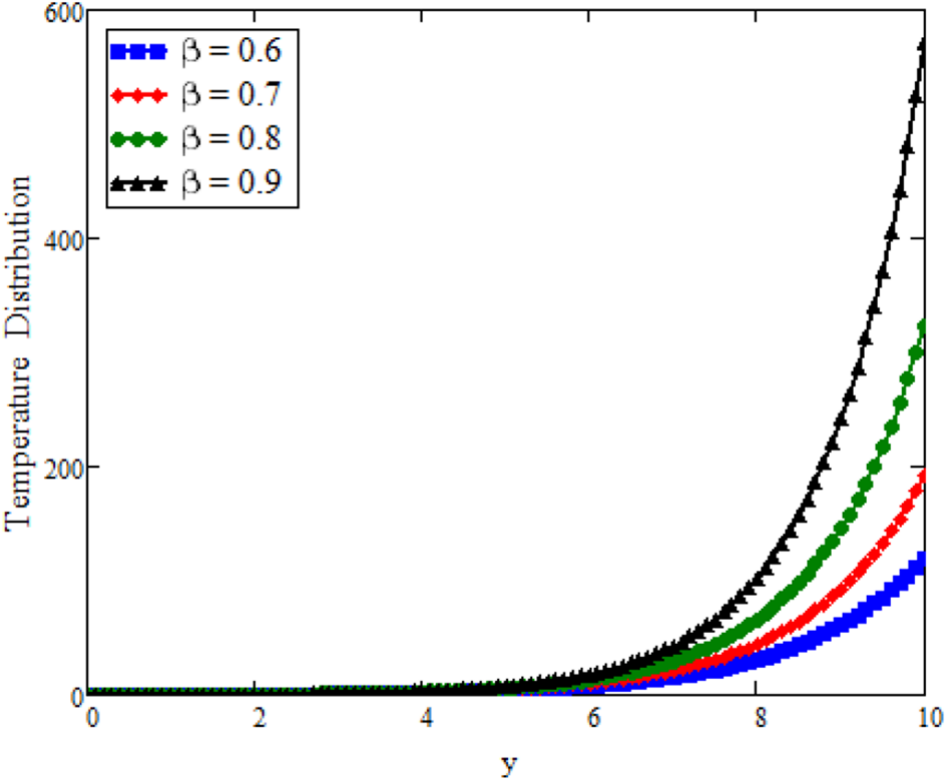
Temperature distribution against y due to $\beta$ for two dimensional graph, when: $t=2$, $\phi _{1}=0.6$, $\phi _{2}=0.6$, $\text {Pr}=5$ and $Re=1$.

**Figure 6:**
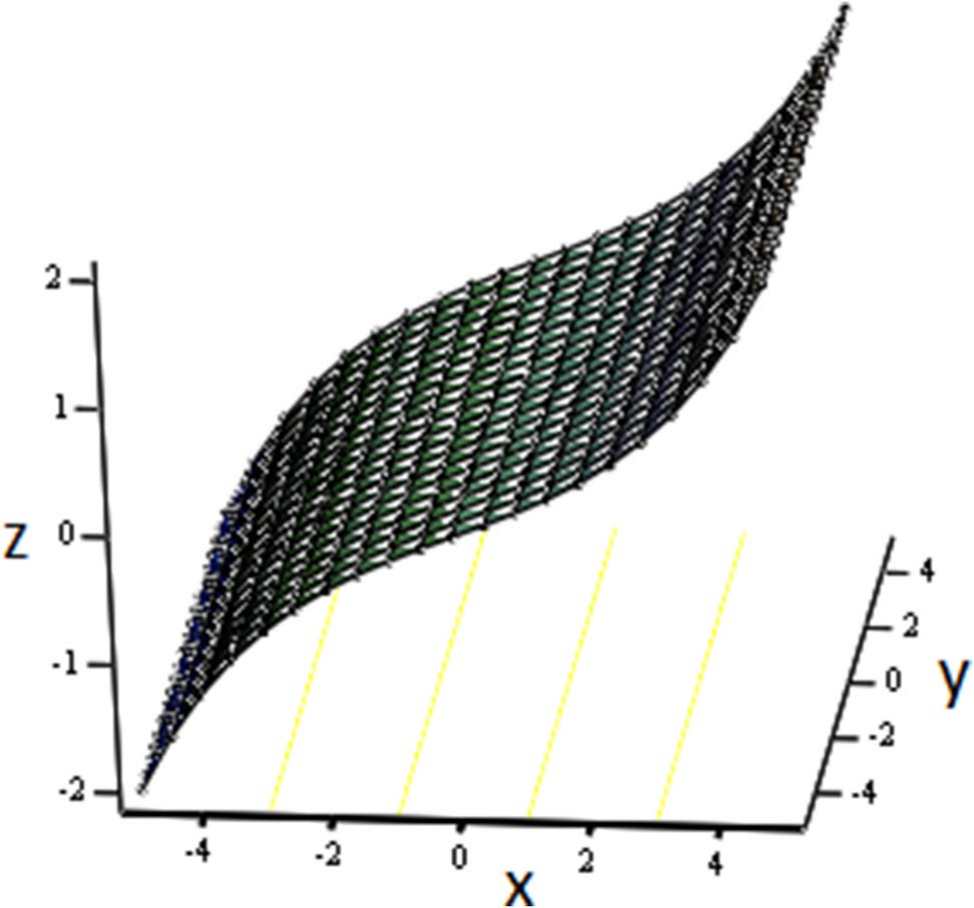
Temperature distribution against y due to $\beta$ for three dimensional graph, when: $t=2$, $\phi _{1}=0.6$, $\phi _{2}=0.6$, $\beta =0.1$, $\text {Pr}=5$ and $Re=1$.

**Figure 7:**
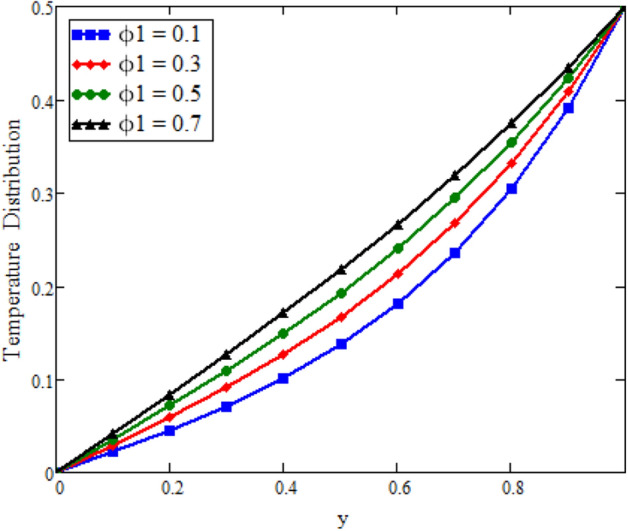
Temperature distribution against y due to $\phi _{1}$ for two dimensional graph, when: $t=0.1$, $\beta =0.4$, $\phi _{2}=0.08$, $\text {Pr}=8$ and $Re=1.5$.

**Figure 8:**
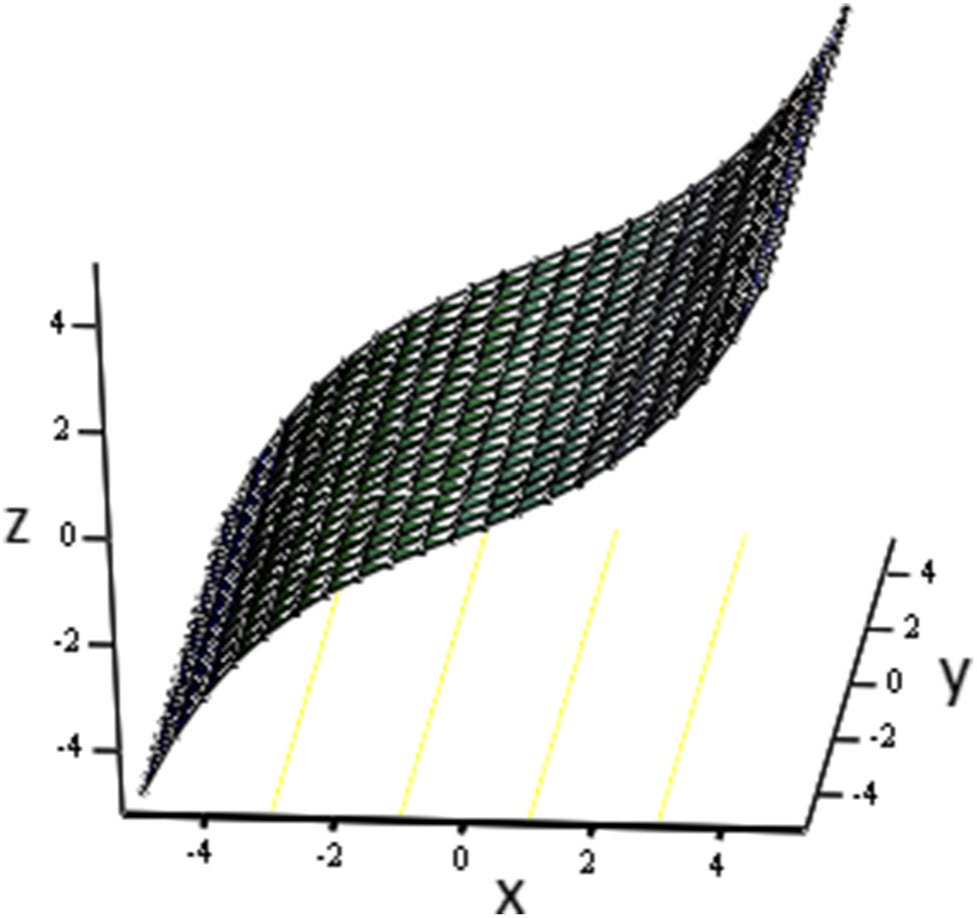
Temperature distribution against y due to $\phi _{1}$ for three dimensional graph, when: $t=0.1$, $\phi _{1}=0.04$, $\phi _{2}=0.08$, $\beta =0.4$, $\text {Pr}=8$ and $Re=1.5$.

**Figure 9:**
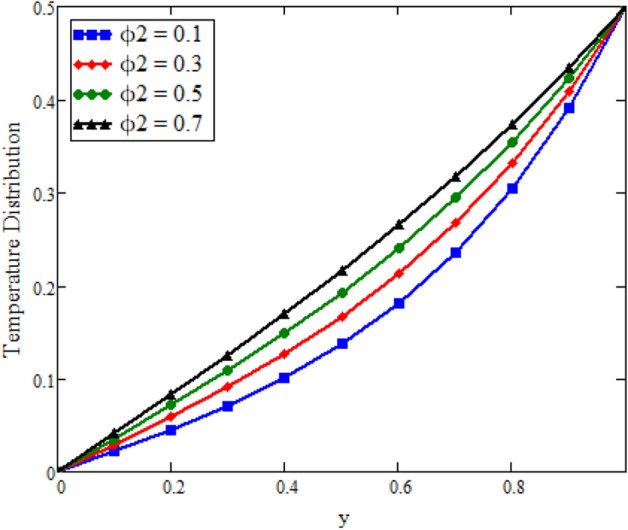
Temperature distribution against y due to $\phi _{2}$ for two dimensional graph, when: $t=0.1$, $\beta =0.4$, $\phi _{1}=0.08$, $\text {Pr}=8$ and $Re=1.5$.

**Figure 10:**
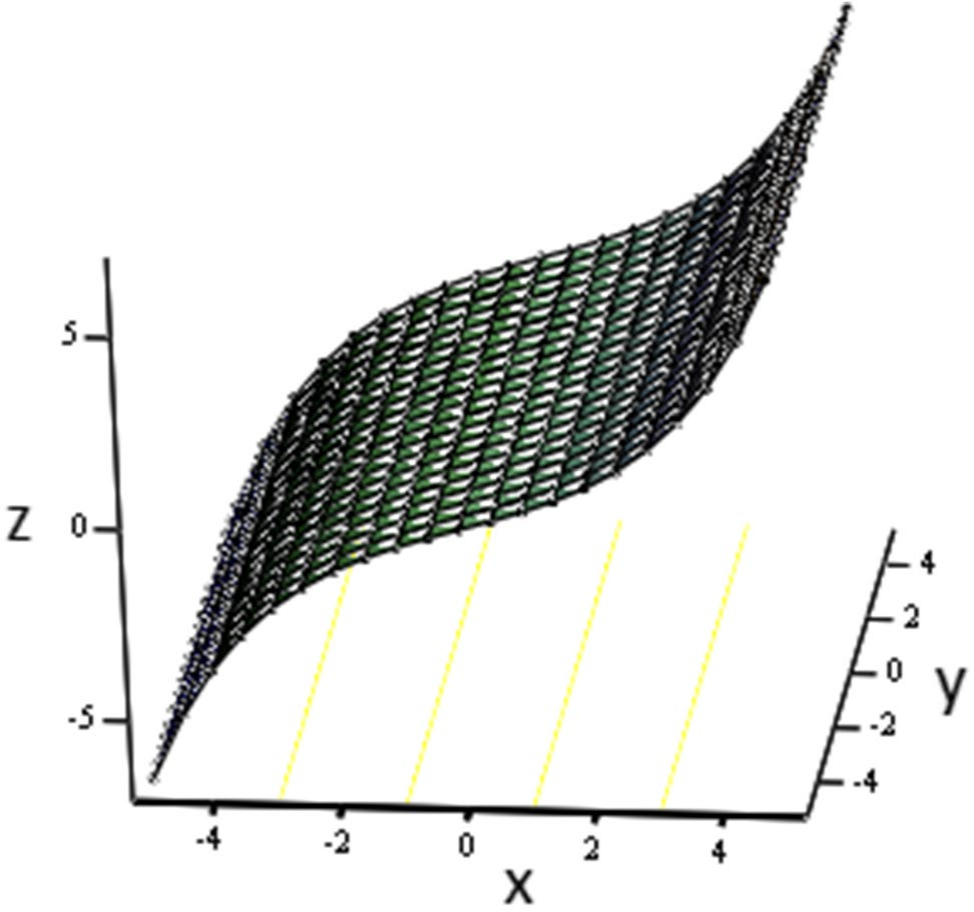
Temperature distribution against y due to $\phi _{2}$ for three dimensional graph, when: $t=0.1$, $\phi _{1}=0.08$, $\phi _{2}=0.08$, $\beta =0.4$, $\text {Pr}=8$ and $Re=1.5$.

**Figure 11:**
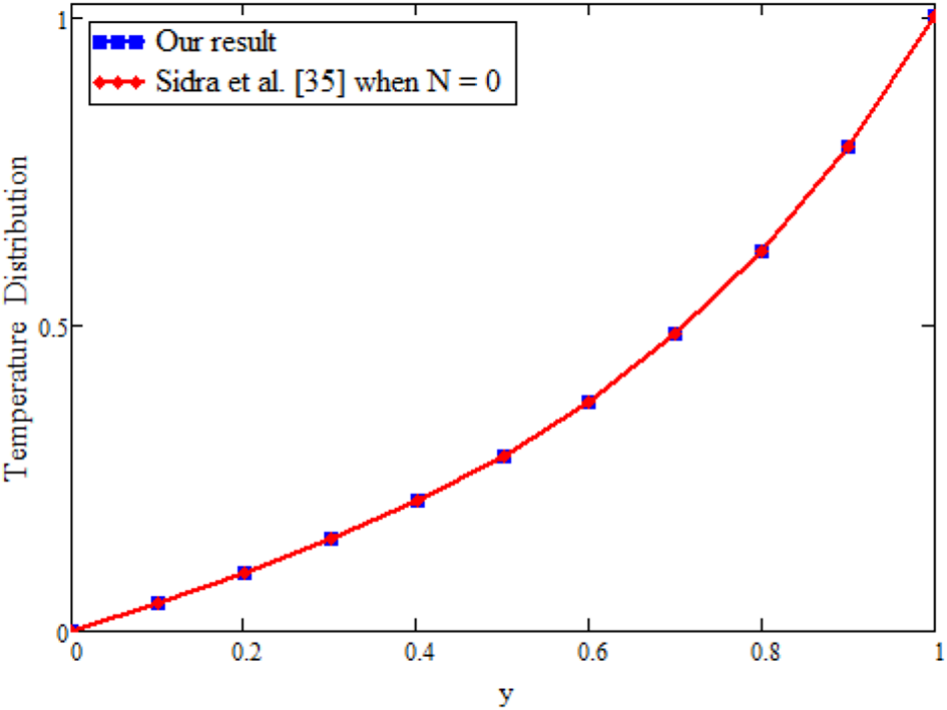
Temperature comparison of our result and Sidra et al.^35^, when: $t=1$, $\beta =0.2$, $\text {Re}=1$, $\text {Pr}=6$, $\phi _{1}=0.04$ and $\phi _{2}=0.04$.

The effects of fractional parameter $\gamma$ on concentration profiles is presented in Fig. 2. The concentration increases as we enhance the values of fractional parameter. Figure 3 represent the three dimensional graph of $\gamma$ for concentration. The concentration comparison with Sidra et al.^35^ is shown in Fig. 4 and both results shows the good agreement with each other. In Fig. 5 by enhancing the values of fractional parameter $\beta$, the temperature increasing. This can be physically justified as when $\beta$ is increased, the momentum and thermal boundary layer decreased and became thinnest at $\beta$ = 1 as a result, the temperature profile decreased. The three dimensional graph of temperature for $\beta$ is shown in Fig. 6. The influence of $\phi _{1}$ and $\phi _{2}$ on temperature profile are studied in Figs. 7, 8 and 9. The temperature profile increases with increase in $\phi _{1}$ and $\phi _{2}$. The is due to the thermal conductivity increasing with the boost of $\phi _{1}$ and $\phi _{2}$ and the fluid showing more heat consequently, of heat transfer increases, which clues to an increase in the temperature profile. Figures 8, 9 and 10 signifies the three dimensional graph of temperature for $\phi _{1}$ and $\phi _{2}$. Figure 11 represents the temperature comparison with Sidra et al.^35^ when N = 0 and both results shows the good agreement.

**Figure 12:**
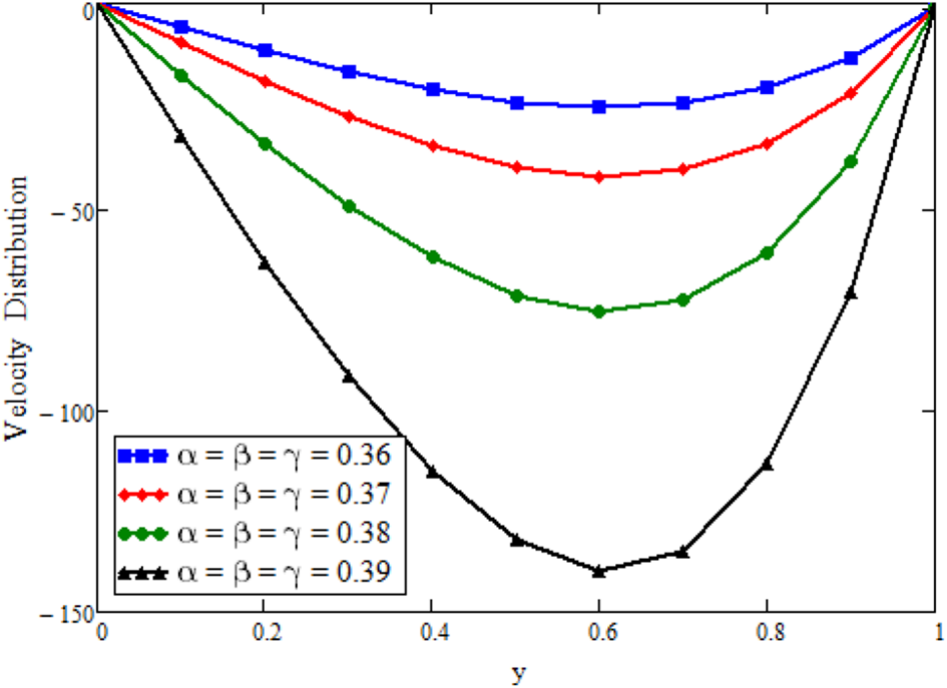
Velocity distribution against y due to equal fractional parameters, when: $t=0.6$, $\phi _{1}=0.04$, $\phi _{2}=0.04$, $\text {Pr}=6$, $\lambda _{0} = 1.2$, $\lambda =0.5$, $\omega =0.5$, $Sc=0.1$, $M=0.5$, $\lambda _{2}=1$, $\text {Gr}=0.0000005$, $\text {Gm}=0.5$ and $\text {Re}=0.0005$.

**Figure 13:**
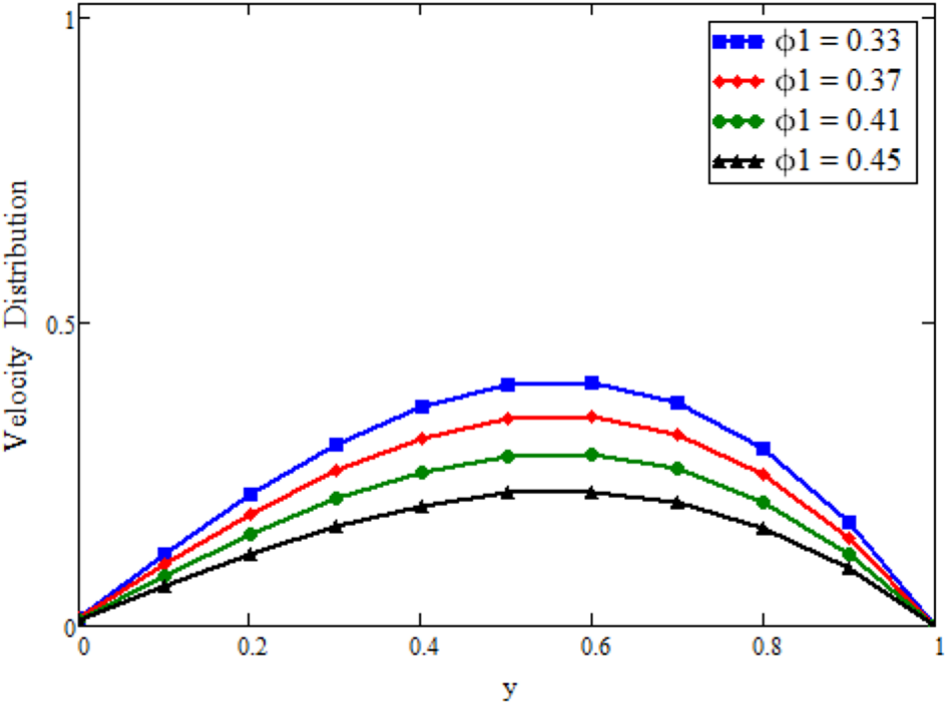
Velocity distribution against y due to $\phi _{1}$, when: $t=0.04$, $\phi _{2}=0.8$, $\text {Pr}=6$, $\lambda _{0} = 1.2$, $\lambda =0.5$, $\omega =0.5$, $Sc=0.1$, $M=0.01$, $\lambda _{2}=2$, $\text {Gr}=0.05$, $\text {Gm}=1$, $\text {Re}=0.1$, $\alpha =0.2$, $\beta =0.2$ and $\gamma =0.2$.

**Figure 14:**
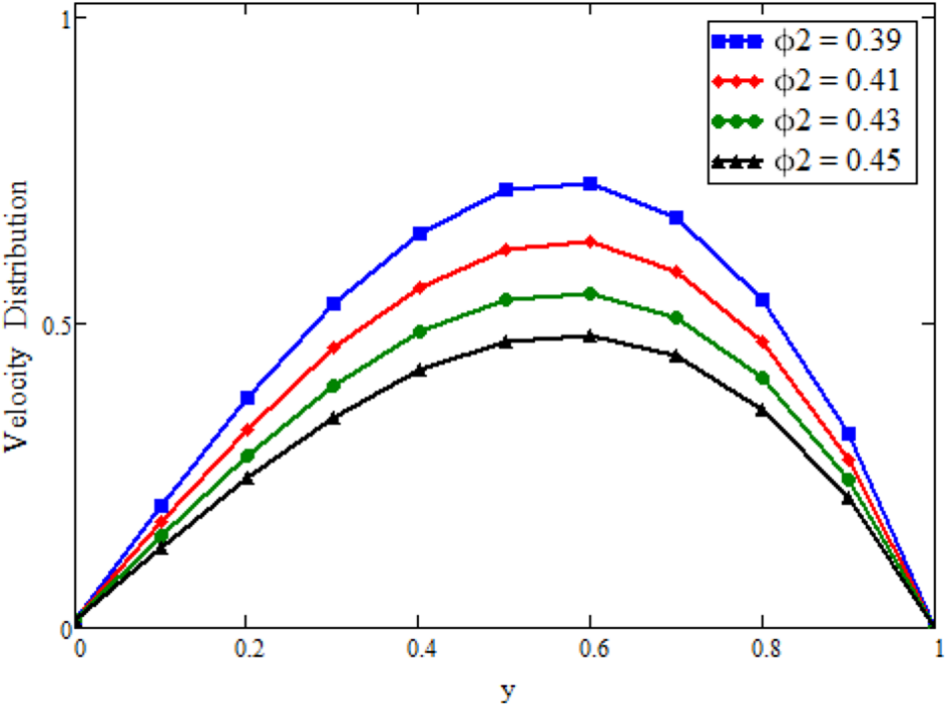
Velocity distribution against y due to $\phi _{2}$, when: $t=0.04$, $\phi _{1}=0.8$, $\text {Pr}=6$, $\lambda _{0} = 1.2$, $\lambda =0.5$, $\omega =0.5$, $Sc=0.1$, $M=1$, $\lambda _{2}=2$, $\text {Gr}=0.05$, $\text {Gm}=1.5$, $\text {Re}=0.1$, $\alpha =0.2$, $\beta =0.2$ and $\gamma =0.2$.

**Figure 15:**
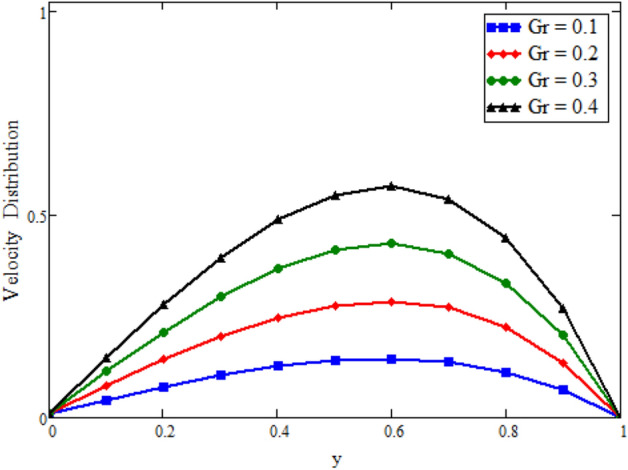
Velocity distribution against y due to $\text {Gr}=0.05$, when: $t=1.7$, $\phi _{1}=0.8$, $\phi _{2}=0.8$, $\text {Pr}=6$, $\lambda _{0} = 1.2$, $\lambda =0.5$, $\omega =0.5$, $Sc=1$, $M=0.5$, $\lambda _{2}=2$, $\text {Gm}=0$, $\text {Re}=1.5$, $\alpha =0.2$, $\beta =0.2$ and $\gamma =0.2$.

**Figure 16:**
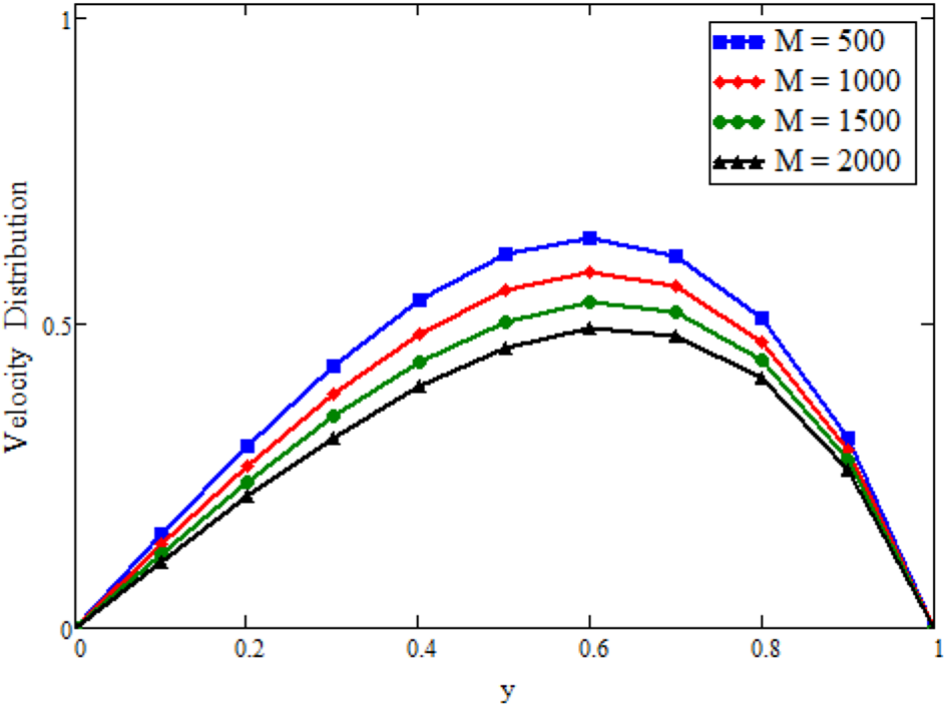
Velocity distribution against y due to *M*, when: $t=1.7$, $\phi _{1}=0.8$, $\phi _{2}=0.8$, $\text {Pr}=6$, $\lambda _{0} = 1.2$, $\lambda =0.5$, $\omega =0.5$, $Sc=0.01$, $\text {Gr}=0.5$, $\lambda _{2}=2$, $\text {Gm}=0$, $\text {Re}=1.5$, $\alpha =0.2$, $\beta =0.2$ and $\gamma =0.2$.

**Figure 17:**
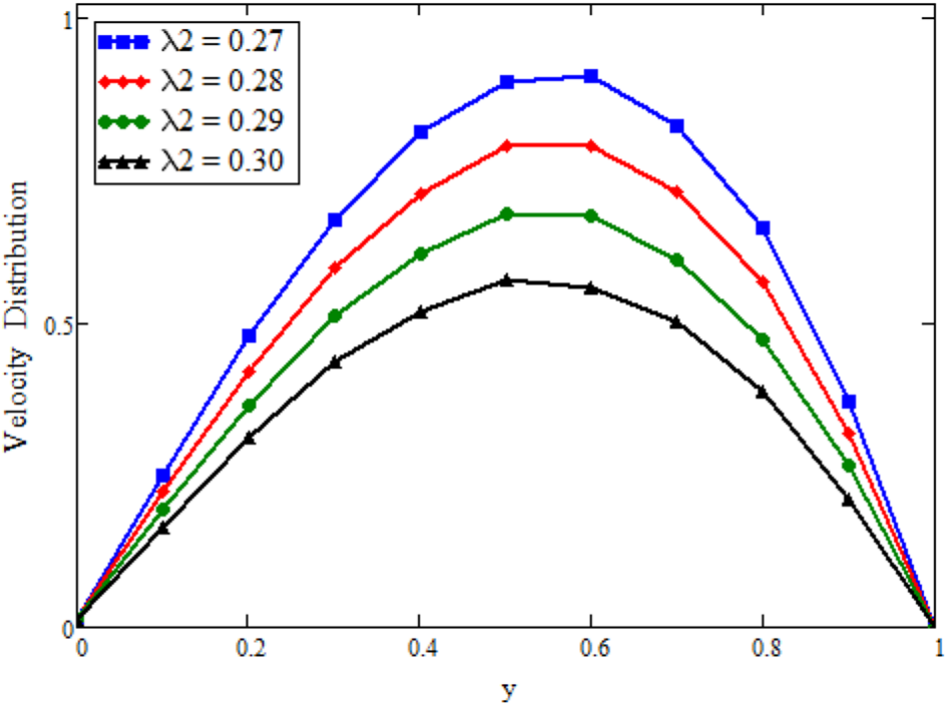
Velocity distribution against y due to $\lambda _{2}$, when: $t=1.7$, $\phi _{1}=0.8$, $\phi _{2}=0.8$, $\text {Pr}=6$, $\lambda _{0} = 1.2$, $\lambda =0.5$, $\omega =0.5$, $Sc=1$, $\text {Gr}=1.95$, $M=0.5$, $\text {Gm}=0.001$, $\text {Re}=1.5$, $\alpha =0.2$, $\beta =0.2$ and $\gamma =0.2$.

**Figure 18:**
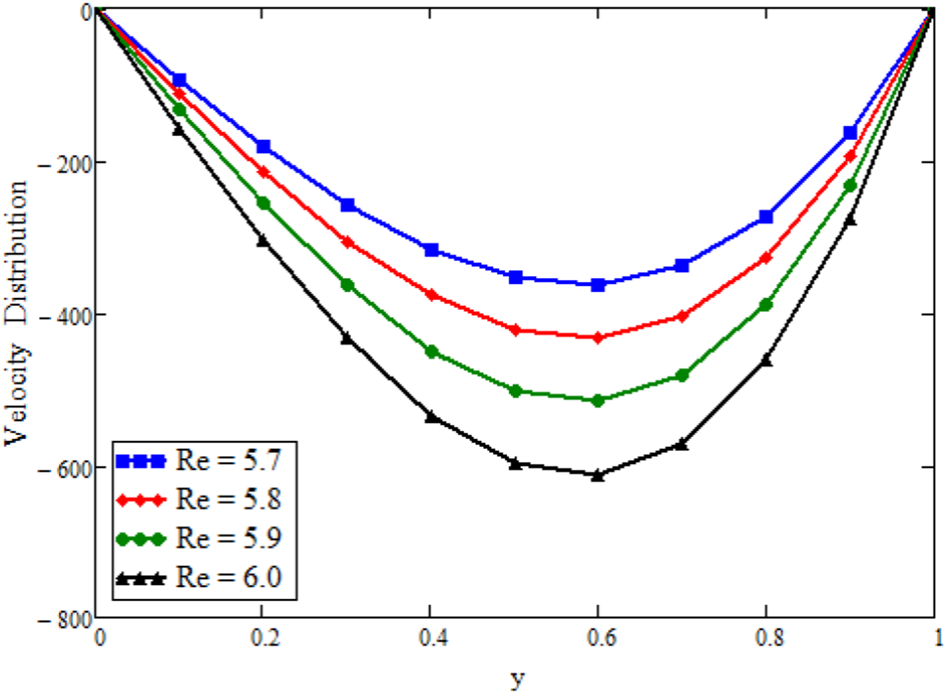
Velocity distribution against y due to $\text {Re}$, when: $t=0.08$, $\phi _{1}=0.8$, $\phi _{2}=0.8$, $\text {Pr}=6$, $\lambda _{0} = 1.2$, $\lambda =0.5$, $\omega =0.5$, $Sc=0.01$, $\text {Gr}=0.1$, $M=0.5$, $\text {Gm}=5$, $\lambda _{2}=1$, $\alpha =0.2$, $\beta =0.2$ and $\gamma =0.2$.

**Figure 19:**
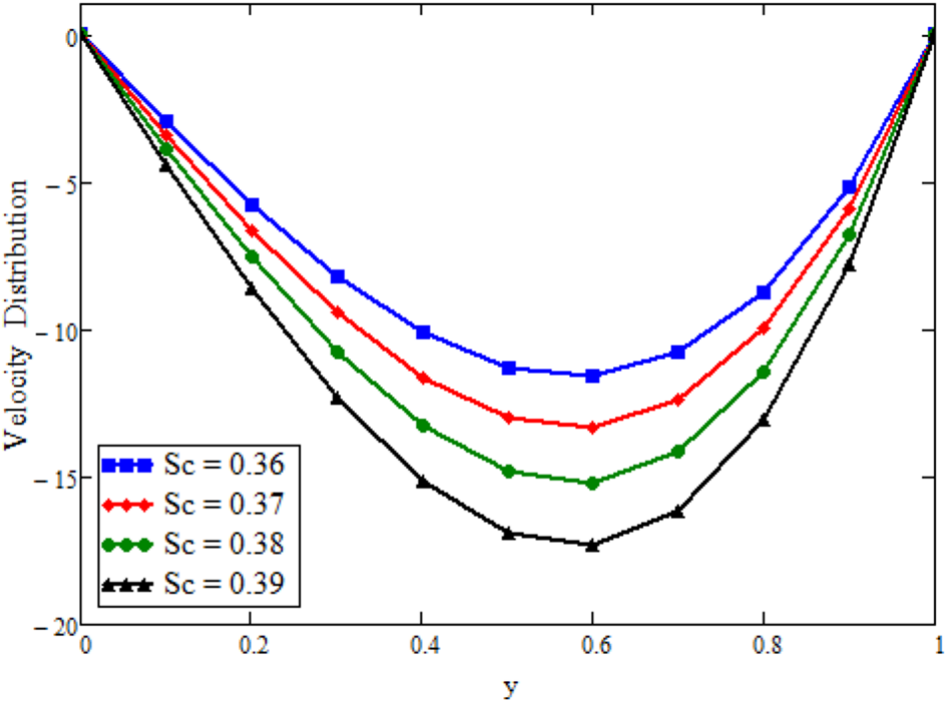
Velocity distribution against y due to *Sc*, when: $t=1.8$, $\phi _{1}=0.8$, $\phi _{2}=0.8$, $\text {Pr}=6$, $\lambda _{0} = 1.2$, $\lambda =0.5$, $\omega =0.5$, $\text {Re}=1.5$, $\text {Gr}=0.1$, $M=0.5$, $\text {Gm}=12$, $\lambda _{2}=2$, $\alpha =0.2$, $\beta =0.2$ and $\gamma =0.2$.

**Figure 20:**
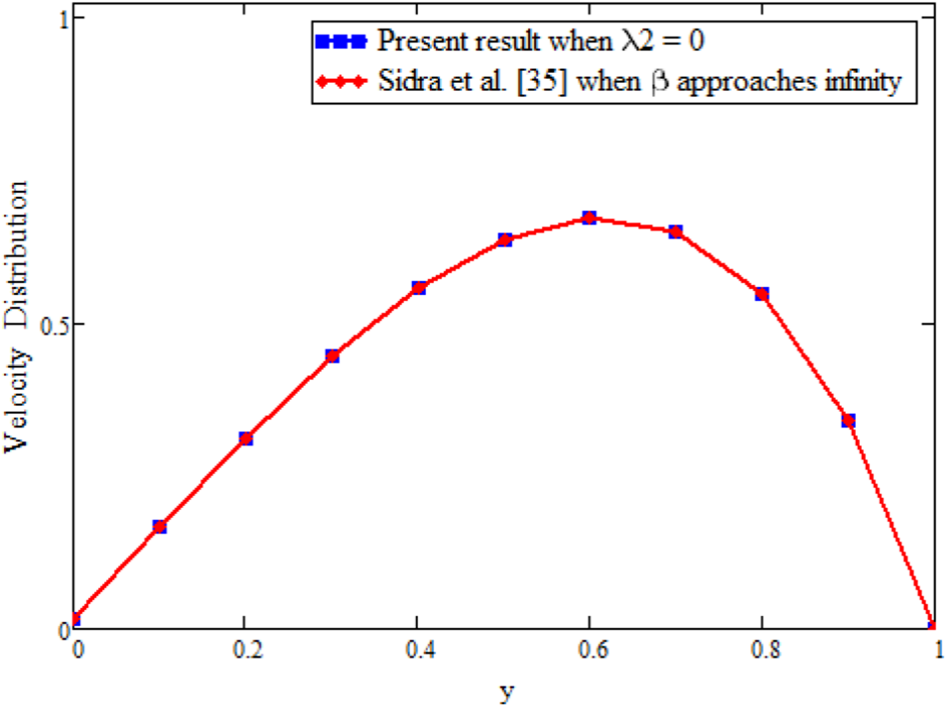
Velocity comparison of our result when $\lambda _{2}=0$ with Sidra et al.^42^ when $\beta =0$.

**Figure 21:**
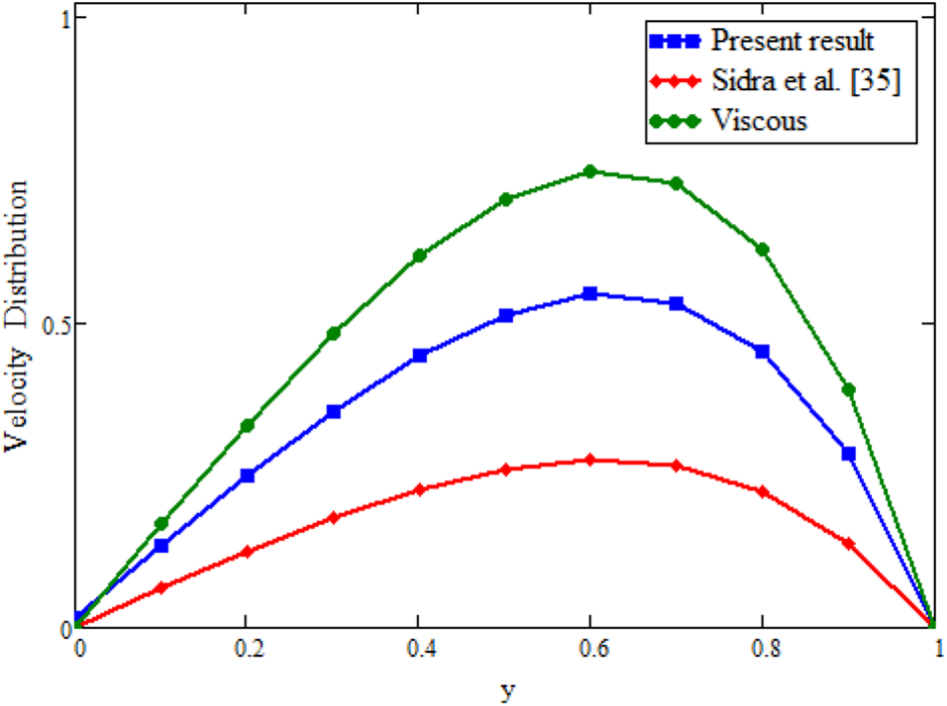
Velocity comparison between Newtonian and non-Newtonian fluids models with fractional derivatives.

Figure 12 is plotted to see the impact of fractional parameters. The fluid velocity reduces as we enhance the values of fractional parameters. This can be physically justified as when we increased fractional parameter, the momentum and thermal boundary layer decreased as a result the velocity profile decreased. The outcome of $\phi _{1}$ and $\phi _{2}$ on the fluid velocity are presented in Figs. 13 and 14. The fluid velocity decreases with increasing $\phi _{1}$ and $\phi _{2}$. This can be physically acceptable as the fluid became more viscous with increasing $\phi _{1}$ and $\phi _{2}$, which clues to a decrease in the fluid velocity. The effect of Grashof number $\text {Gr}$ is studied in Fig. 15. In this figure, it is detected that for larger values of $\text {Gr}$ the the fluid velocity shows an increasing trend. This is because when $\text {Gr}$ is increased the buoyancy forces become stronger due to which more convection takes place. As a result, the velocity profile increases. The velocity reduces as we rise the Magnetic parameter M in Fig. 16. Physically, it can occurs that answerable the drag force, which effects on the velocity field that faces the fluid motion, affects in reducing the velocity. Figure 17 shows that the velocity is a deceasing function of relaxation parameter $\lambda _{2}$ as we increased the magnitude of velocity. Figure 18 is presented the influence of Reynolds number Re and it can be seen that fluid velocity near the plate is maximum and decreases in its free stream region, as we increased the values of Reynolds number fluid velocity decreases. It is due to the fact that The Reynolds number (Re) helps predict flow patterns in different fluid flow situations. At low Reynolds numbers, flows tend to be dominated by laminar (sheet-like) flow, while at high Reynolds numbers flows tend to be turbulent. It is found that by increasing the values of Re the fluid velocity is also decreases for all fractional parameters. This happened due to the fact that Re is a dimensionless number usually appear in fluid dynamics which characterized the flow behavior. It is ratio between inertial force and viscous force. It is the relative strength of inertial forces to viscous forces. The relative strength of these two actions their ratio does have a lot of influence on how the fluid flow behaves. Therefore, viscous force is more dominant is this case and responsible to slow down the fluid flow as well as reduce the boundary layer thickness between the models. Figure 19 depicts the impact of Schmidt number Sc on fluid velocity. By observing the figure we can see the increasing value of the Schmidt number with the decrease in the velocity profile due to the decrease in the molecular diffusivity, which turns to a decrease in the concentration and the thickness along the boundary layers of velocity. In order to check the validity of the present results of Maxwell fluid with the existing literature, we presented Fig. 20. It is found that when Casson parameter in^35^ approaches to infinity and the relaxation parameter in the Maxwell fluid approaches to zero, both the obtained results are in good agreement. Further, an other comparison between the different fluids models like Maxwell, Casson and viscous fluid we plotted Fig. 21 and it is clear that viscous fluid has higher velocity than Maxwell and Casson fluids. It is due to the reason that viscous fluid has less viscosity than others thats why it flows with larger velocity.

**Figure 22:**
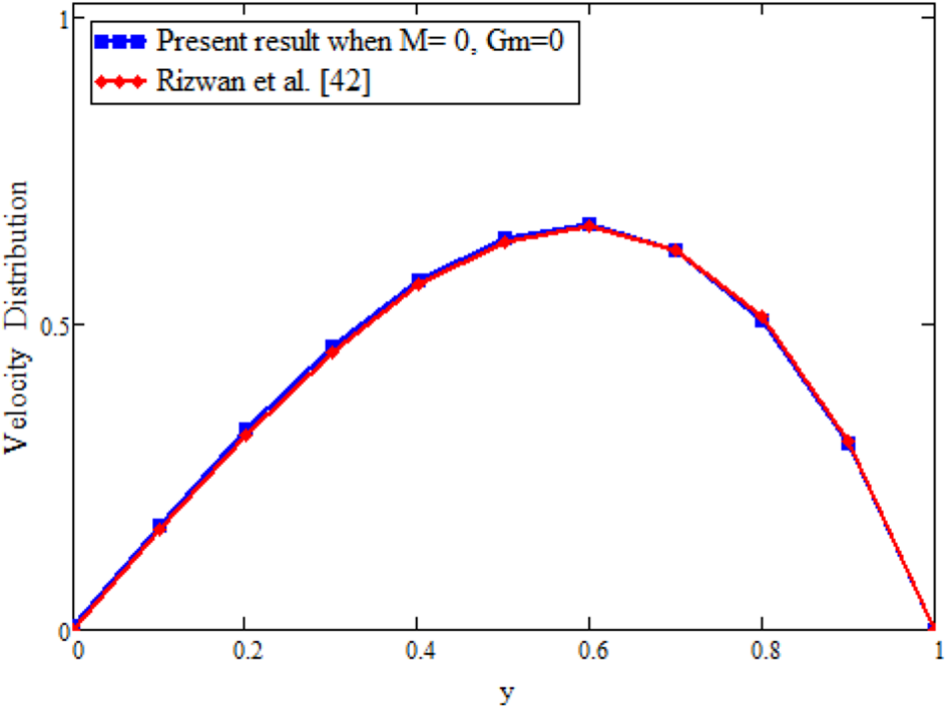
Velocity comparison of our result with Rizwan et al.^42^, when: $M=0$ and $\text {Gm}=0$.

**Figure 23:**
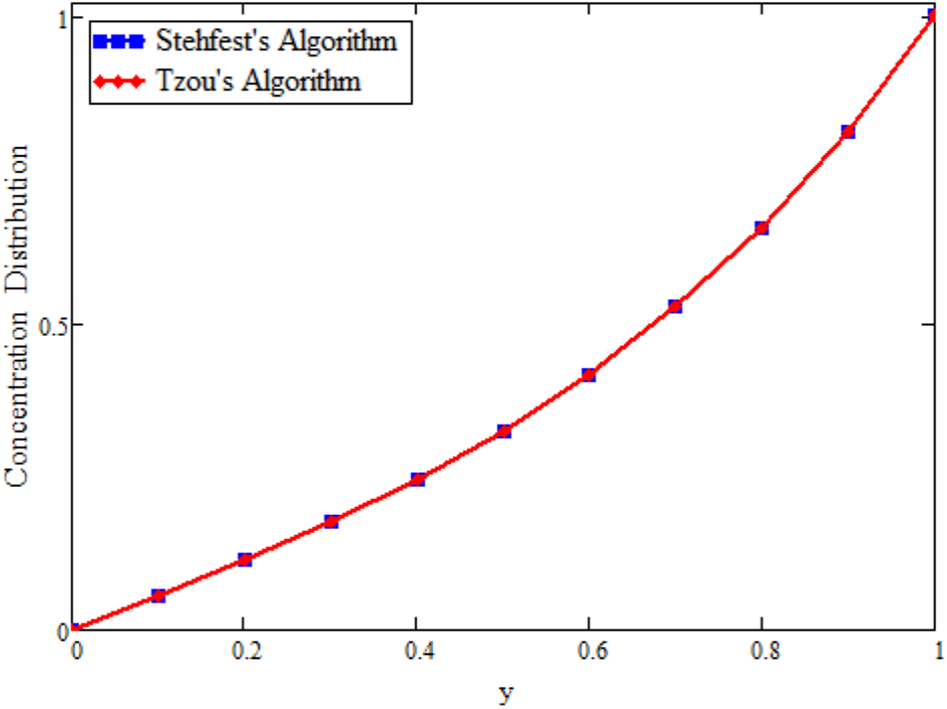
Inverse Laplace transform of the concentration profile by Stehfest’s and Tzou’s algorithms.

**Figure 24:**
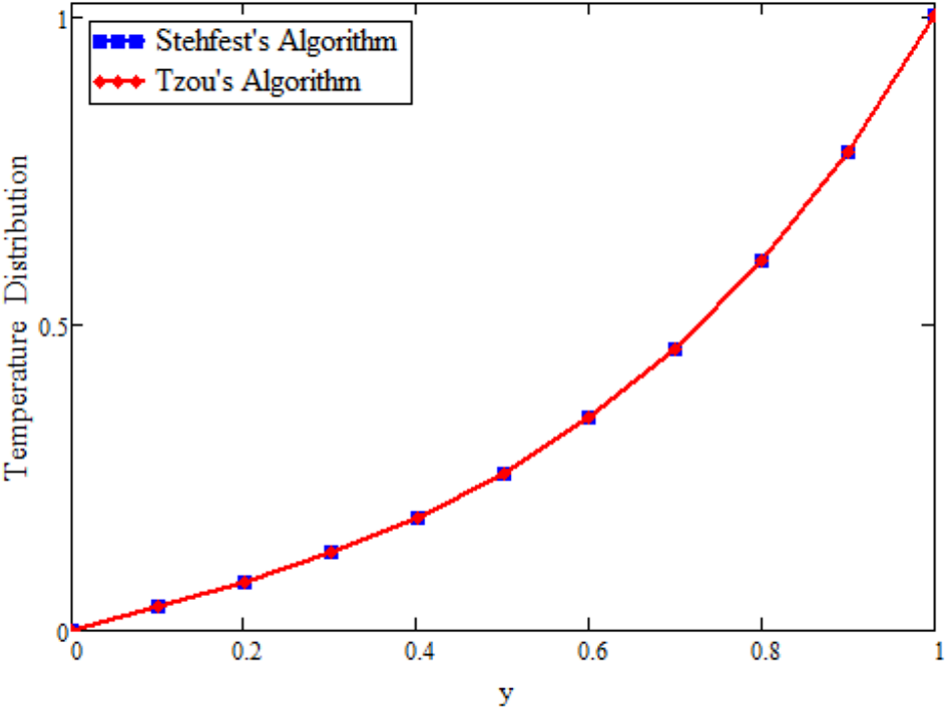
Inverse Laplace transform of the temperature profile by Stehfest’s and Tzou’s algorithms.

**Figure 25:**
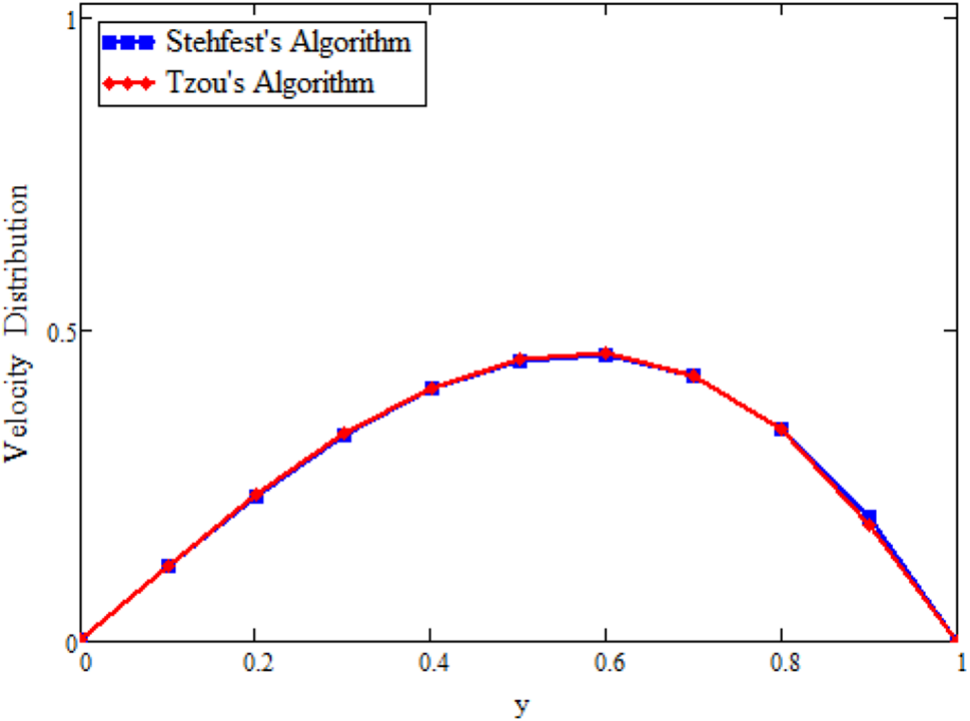
Inverse Laplace transform of the velocity profile by Stehfest’s and Tzou’s algorithms.

Figure 22 shows the comparison of velocity in the absence of magnetohydrodynamics $M = 0$ and mass transfer $\text {Gm} = 0$, and compared with Rizwan et al.^42^ it is found that both results show the same behavior. In order to support the inversion algorithms of Laplace transform for temperature, concentration and velocity fields we have plotted Figs. 23, 24 and 25 and found that they are in good agreement.

## Conclusions

In this paper we discuss the Maxwell hybrid nanofluids (*Cu* and $\mathrm{Al}_{2}\mathrm{O}_{3}$) due to pressure gradient into rectangular region using Caputo time fractional operator. Exact analytical solutions are setteled for concentration, temperature and velocity profiles via the Laplace transform technique. The influence of various parameters are numerically studied through graphs and discuss physically. The major points extracted from this study are as follows:

1. Temperature and concentration showed dual behavior for fractional parameters $\beta$ and $\gamma$ for small and large time due to power law nature of the kernel.
2. Increasing the values of nanoparticles volume fraction $\phi _{1}$ and $\phi _{2}$, consequently increases the temperature and decreases the velocity.
3. The values of fractional parameters Reynolds number $\text {Re}$, Schmdit number Sc, and magnetic parameter $\text {M}$ increases, then velocity decreases.
4. We have compared the present results with the existing models and found that they are in good agreement.
5. In comparison of Newtonian and non-Newtonian fluids models it is found that viscous fluid faster than Maxwell and Casson fluids.
